# Scaffold Proteins in Fibrotic Diseases of Visceral Organs

**DOI:** 10.3390/biom15030420

**Published:** 2025-03-16

**Authors:** Piaopiao Sun, Liliang Yang, Keqing Yu, Jing Wang, Jie Chao

**Affiliations:** Jiangsu Provincial Key Laboratory of Critical Care Medicine, Zhongda Hospital, Department of Physiology, School of Medicine, Southeast University, Nanjing 210009, China

**Keywords:** scaffold proteins, fibrosis, signal transduction

## Abstract

Fibrosis, characterized by excessive extracellular matrix (ECM) deposition, disrupts tissue architecture and impairs organ function, ultimately leading to severe health consequences and even failure of vital organs such as the lung, heart, liver, and kidney. Despite significant advances in understanding the molecular mechanisms underlying fibrosis, effective therapeutic options remain limited. Emerging evidence highlights scaffold proteins as critical regulators in the progression of fibrosis. These multifunctional proteins serve as molecular platforms that organize and coordinate key signaling pathways—including those governing ECM remodeling, cytoskeletal organization, and cell migration—thereby integrating both profibrotic and antifibrotic signals. Their pivotal role in linking mechanotransduction, inflammatory, and developmental signals offers a unique therapeutic window, as targeted interventions (e.g., small-molecule inhibitors, peptides, biologics, and gene therapy) are emerging to modulate these pathways. This review synthesizes recent findings on scaffold protein functions across multiple organs and discusses novel therapeutic strategies to manage and potentially reverse fibrosis.

## 1. Introduction

Fibrosis is a major contributor to global morbidity and mortality, marked by excessive extracellular matrix (ECM) accumulation that disrupts tissue architecture and compromises organ function. This pathological process arises from persistent, repetitive injuries that overwhelm normal tissue repair mechanisms, triggering fibroblast activation and pathological collagen deposition. The initiation and amplification of fibrogenesis involve complex interactions among various cell populations: epithelial and endothelial cells undergoing mesenchymal transitions, as well as immune cells like macrophages that orchestrate inflammatory responses. By secreting transforming growth factor-beta (TGF-β) and other profibrotic mediators, macrophages activate fibroblasts and exacerbate matrix production. Increased vascular permeability further facilitates immune cell infiltration into tissues, fueling a cycle of injury and repair that drives fibrosis in organs such as the lung, heart, liver, and kidney. Despite some evidence that fibrosis can be halted or even reversed if the underlying cause is removed, current therapeutic options remain limited. This underscores an urgent need to unravel the molecular mechanisms driving fibrosis and identify novel targets for intervention.

Scaffold proteins have recently emerged as critical regulators in fibrosis pathogenesis. Traditionally regarded as passive structural adaptors that tether proteins together, scaffolds are now recognized for actively modulating key signaling pathways implicated in fibrotic disease. These multifunctional proteins act as molecular platforms that organize and integrate diverse signaling molecules, ensuring precise spatial and temporal regulation of cellular responses. They thereby coordinate fundamental processes like ECM remodeling, cytoskeletal organization, and cell differentiation that are central to fibrosis progression. Given their nodal position in signaling networks, scaffold proteins present an attractive yet challenging class of therapeutic targets. This review addresses how scaffold proteins function as pivotal regulators in the signaling networks underlying fibrosis. In particular, we organize the discussion by major profibrotic signaling pathways—including TGF-β, inflammatory/immune, Wnt/β-catenin, mechanotransduction, and other stress-related pathways—to highlight shared mechanisms and context-specific nuances (Table 1 and Table 2). By examining scaffold protein roles across these pathways, we aim to illustrate their integrative functions in fibrosis and their potential as targets for therapeutic modulation.

## 2. Materials and Methods

In PubMed (https://pubmed.ncbi.nlm.nih.gov/), our search strategy was as follows: First, we searched for all scaffold proteins related to fibrosis using the query: (fibrosis[Title/Abstract] AND (scaffold[Title/Abstract] OR adaptor[Title/Abstract]) AND (y_5[Filter])) AND (protein[Title/Abstract]). Additionally, we referred to the human scaffold proteins summarized by Han et al. in 2016 [36] and identified those related to fibrosis through PubMed. However, during our review process, we found that not all scaffold proteins influencing fibrosis exert their effects through their scaffold function. Nevertheless, as long as they are involved in organ fibrosis, we included them in this review.

## 3. Overview of Scaffold Proteins

Definition and Characteristics: Scaffold proteins are broadly recognized as proteins that coordinate multiple signaling components (e.g., enzymes, receptors) to enhance pathway efficiency and specificity. Hall et al. define them as proteins associating with ≥2 partners to optimize signaling outcomes [37], while Langeberg et al. emphasize three core features [38]: (1) multivalent binding to tether cascade components, (2) non-catalytic spatial organization to concentrate enzymes, and (3) modular frameworks enabling bidirectional pathway control. Bao et al. further characterize scaffolds as structural, non-enzymatic regulators that boost signaling via localized concentration, molecular orientation, and allosteric modulation [39]. However, the term lacks a universal definition, and its usage remains context-dependent. In this study, scaffold protein selection follows criteria detailed in the Section 2 to ensure consistency. For this review, we focus on proteins that clearly meet these scaffolding criteria; i.e., they possess multiple interaction domains to assemble signaling complexes. Each selected protein has a documented role as a molecular platform coordinating fibrogenic signals, as detailed below.

Biological Roles of Scaffolds: Since the 1980s, the understanding of scaffold proteins has undergone a remarkable transformation, evolving from their initial perception as static “assembly platforms” to being recognized as dynamic regulators of cellular signaling. Early studies focused on how scaffold proteins cluster kinases, phosphatases, and receptors, as exemplified by Grb2 in the Ras/MAPK pathway [40,41,42]. By the 1990s, A-kinase anchoring proteins (AKAPs) illustrated how scaffolds confer spatial specificity: AKAPs tether protein kinase A (PKA) to defined subcellular locales, thus confining cAMP signals to discrete microdomains [43,44]. Advances in proteomics and imaging later revealed that some scaffolds drive the formation of membraneless organelles via phase separation, adding another layer of spatial control in cell signaling [45,46]. Importantly, scaffold proteins are found in all cellular compartments—at the plasma membrane, in the cytosol, and in the nucleus—orchestrating signaling pathways in a location-dependent manner. For example, caveolin-1 (Cav1) at membrane caveolae acts as a scaffold that organizes specific lipids and receptors (e.g., eNOS, TGF-β receptors), thereby modulating nitric oxide and TGF-β signaling in fibrosis. Annexin A2, another membrane-associated scaffold, links phospholipids to actin, helping organize membrane rafts and cytoskeletal dynamics crucial for cell signaling [47]. In the cytoplasm, IQGAP1 binds multiple partners (actin, Rac, MEK, β-catenin, etc.), influencing cell morphology and motility while integrating signals from growth factor and Wnt pathways [48,49]. Classic signaling scaffolds in yeast (Ste5 in the MAPK mating pathway [50,51]) and neurons (Shank proteins coupling receptors to the actin cytoskeleton [52,53]) further exemplify the conserved role of scaffolds in pathway insulation and efficiency. In the nucleus, scaffolds such as promyelocytic leukemia protein (PML) and BRD4 organize transcriptional complexes (as discussed later), while nuclear pore scaffolds (nucleoporins) maintain nucleocytoplasmic transport and signaling gradients. Overall, scaffold proteins enhance signaling specificity by coordinating the assembly of pathway components in space and time, thereby amplifying intended outputs and limiting inappropriate crosstalk.

Scaffold Proteins in Fibrosis: Fibrosis involves the dysregulation of multiple signaling pathways, with TGF-β, inflammatory/immune signals, Wnt/β-catenin, mechanotransduction, and metabolic stress pathways all playing pivotal roles. Scaffold proteins serve as multifunctional nodes within these cascades, simultaneously binding signaling enzymes, receptors, and substrates to ensure that fibrogenic signals are properly relayed. By tethering upstream stimuli to downstream effectors, scaffolds can accelerate profibrotic signaling (e.g., by bringing kinases and substrates into proximity) or attenuate signals (e.g., by sequestering transcription factors away from DNA). Because they can modulate pathway intensity like a rheostat, scaffolds often determine whether transient injury signals tip into sustained fibrotic responses. Below, we examine key scaffold proteins operating in the major fibrosis-associated pathways. Each section begins with a brief background on the pathway’s role in fibrosis, followed by specific scaffold proteins that influence that pathway. We highlight how these scaffolds meet the criteria above and describe their contributions to fibrotic disease. Redundant discussion of a given scaffold is avoided by cross-referencing between sections; for instance, an integrin’s role in mechanotransduction (Section 4.4) is mentioned briefly in the TGF-β section as needed rather than repeated in full.

## 4. Scaffold Proteins as Signaling Hubs in Fibrosis

### 4.1. TGF-β Signaling Pathway

Pathway Background: The TGF-β signaling pathway is a central driver of fibrosis in virtually all organs. TGF-β ligands (especially TGF-β1) are typically released in a latent form bound to the ECM, and when activated they bind TGF-β receptors on target cells to trigger intracellular SMAD signaling. In fibroblasts, excessive or prolonged TGF-β signaling induces differentiation into myofibroblasts, highly synthetic, contractile cells that produce large amounts of collagen and other ECM components. TGF-β/Smad signaling also upregulates genes like α-smooth muscle actin (α-SMA) and connective tissue growth factor (CTGF) that perpetuate the fibrotic phenotype. Aberrant TGF-β activity, whether due to chronic injury or genetic predisposition, leads to pathological ECM deposition, tissue stiffening, and loss of organ function. Given its master regulator role, the TGF-β pathway is tightly controlled at multiple levels, including ligand activation, receptor complex formation, Smad nuclear translocation, and transcriptional regulation. Scaffold proteins influence each of these steps—from controlling the availability of active TGF-β, to organizing receptor–Smad complexes, to modulating Smad-driven gene expression—thereby exerting significant control over fibrogenesis.

Integrins—Activating Latent TGF-β via ECM Mechanosensing: A key class of scaffold-related proteins regulating TGF-β in fibrosis are the integrins. Integrins are transmembrane receptors that physically connect the ECM to the cytoskeleton and can function as mechanotransducers. Certain integrin heterodimers (e.g., αvβ6, αvβ8) are known to bind the latent TGF-β complex and activate it by exerting mechanical force, thus increasing local TGF-β availability in injured tissues. In addition, integrins form focal adhesion complexes that cluster signaling molecules. For example, the α6β1 integrin in lung fibrosis acts as a mechanosensitive scaffold that couples ECM stiffness to TGF-β signaling. In idiopathic pulmonary fibrosis (IPF), α6β1 integrin is upregulated in regions of basement membrane stiffening and co-localizes with TGF-β complexes. Through its cytoplasmic interactions, α6β1 integrin stabilizes TGF-β ligands at the cell surface and promotes matrix metalloproteinase (MMP)-2 mediated degradation of basement membrane collagen-IV, facilitating invasive migration of fibroblasts into the interstitium [1]. This not only enhances TGF-β ligand activation but also feeds a cycle of matrix remodeling and fibrosis. Genetic ablation or antibody blockade of α6 integrin in experimental lung fibrosis significantly reduces myofibroblast infiltration, ECM deposition, and tissue stiffness, underscoring its critical role as a scaffold linking mechanical cues to TGF-β activation. Thus, integrins fulfill scaffold functions by bringing together ECM-bound TGF-β and cell signaling machinery; their activation of latent TGF-β represents a primary control point in fibrosis initiation. Integrins as mechanotransduction scaffolds are further discussed in Section 4.4.

Hic-5 (TGFB1I1)—Focal Adhesion Scaffold Amplifying TGF-β Signals: Hic-5 is a TGF-β1-inducible focal adhesion adaptor protein that exemplifies how scaffolds can amplify downstream signaling. Hic-5 (also called Hic5 or TGFB1I1) is localized at focal adhesions and has multiple protein interaction domains (e.g., LIM domains), allowing it to scaffold signaling complexes. In fibrosis, Hic-5 expression is upregulated by TGF-β and by oxidative stress. Notably, Hic-5 binds to Smad7 (an inhibitory Smad) in the cytoplasm, sequestering Smad7 away from the TGF-β receptor complex [2]. This frees the receptor to phosphorylate Smad2/3 more efficiently. Hic-5 thereby enhances TGF-β/Smad signaling, effectively functioning as a positive regulator by tethering an inhibitor (Smad7) and preventing it from attenuating the pathway. In hepatic fibrosis models, Hic-5 levels increase in proportion to disease severity [3]. Hic-5 interaction with Smad7 promotes prolonged Smad2 phosphorylation and upregulates lysyl oxidase (LOX), an enzyme that crosslinks collagen fibers and increases matrix stiffness. Through this scaffolding mechanism, Hic-5 creates a feed-forward loop: more TGF-β signaling leads to more ECM crosslinking, which can further activate TGF-β. Importantly, therapeutic silencing of Hic-5 has shown antifibrotic effects. In a metabolic dysfunction-associated steatohepatitis (MASH) model of advanced liver fibrosis, antisense oligonucleotides (ASOs) against Hic-5 significantly reduced α-SMA expression and collagen deposition, leading to attenuated fibrosis and even improvement of liver steatosis [4]. These results validate Hic-5 as a bona fide scaffold protein driving TGF-β-mediated fibrosis and highlight that disrupting its scaffold function can dampen fibrotic signaling.

Ski—Nuclear Scaffold and TGF-β/Smad Co-repressor: Ski is an example of a nuclear scaffold protein that negatively regulates TGF-β signaling. Ski (the Ski proto-oncogene) resides in the nucleus and can bind Smad2/3-Smad4 complexes, serving as a co-repressor that prevents Smads from activating TGF-β target genes. By tethering Smads and recruiting transcriptional repressors, Ski effectively silences TGF-β-driven gene expression. In the context of fibrosis, Ski plays a protective, antifibrotic role. For instance, in mouse models of cardiac fibrosis after myocardial infarction, Ski expression is dynamically regulated. Loss of Ski following injury leads to enhanced TGF-β/Smad activity and exacerbates fibrosis, whereas maintaining or overexpressing Ski attenuates fibrotic remodeling [5,6]. Mechanistically, Ski’s scaffold function extends beyond simply sequestering Smads; it also interfaces with other pathways. Recent evidence suggests Ski interacts with the Hippo pathway effector TAZ via the adaptor LIMD1, promoting TAZ degradation and thereby inhibiting profibrotic gene programs. In summary, Ski serves as a scaffold that ties together Smad proteins and components of the transcriptional machinery to restrain TGF-β signaling. Its downregulation in injured tissues removes a critical brake on TGF-β, tilting the balance toward fibrogenesis. This has prompted interest in therapeutic strategies to enhance Ski or mimic its function to counteract TGF-β in fibrosis.

IQGAP1—Cross-Pathway Cytoskeletal Scaffold Modulating TGF-β: IQGAP1 is a versatile cytosolic scaffold that integrates signals from the actin cytoskeleton with multiple pathways, including TGF-β [25,49]. It interacts with a spectrum of proteins (actin filaments, Rac1/Cdc42 GTPases, MAPK components, β-catenin, etc.), positioning it as a nodal hub. In fibrosis, IQGAP1 has a context-dependent role. Under homeostatic conditions, IQGAP1 appears to provide a tonic restraint on fibroblast activation—possibly by organizing signaling complexes that maintain cytoskeletal dynamics and prevent excessive contractile stress fiber formation. In idiopathic pulmonary fibrosis, however, IQGAP1 expression is paradoxically diminished in lung fibroblasts and myofibroblasts. Loss of IQGAP1’s scaffold function in this setting may contribute to unchecked fibrotic activation. Indeed, IQGAP1 knockout mice develop exacerbated pulmonary fibrosis in some studies, but others have reported the opposite trend. Notably, one study found that genetic ablation of IQGAP1 resulted in attenuated TGF-β production and signaling, reduced α-SMA expression, and impaired contractility of fibroblasts, ultimately mitigating fibrosis progression [26]. This suggests that IQGAP1’s role is complex: it might normally scaffold a balance of pro- and antifibrotic signals, and its removal can disrupt fibroblast function in ways that either promote or hinder fibrosis depending on context. What is clear is that IQGAP1 directly binds components of the TGF-β pathway (and others like Wnt/β-catenin), influencing their activity. Thus, IQGAP1 exemplifies a cross-pathway scaffold protein that links TGF-β signaling with cytoskeletal remodeling. Further research is needed to fully delineate why IQGAP1 deficiency can be protective in some fibrotic models, but its ability to modulate multiple pathways simultaneously underscores the importance of scaffold proteins as integrators in fibrosis.

SHARPIN—Scaffold of TGF-β Receptor Complexes: SHARPIN (SHANK-associated RH domain interacting protein) is an intracellular adaptor protein originally known for roles in NF-κB signaling and immune functions, but it also impacts TGF-β signaling in fibrosis. SHARPIN contains multiple interaction domains and can act as a scaffold at the cell membrane and in the cytosol. In cardiac fibrosis, SHARPIN was found to bind and stabilize components of the TGF-β receptor signaling complex, thereby enhancing downstream Smad signaling. This leads to increased myofibroblast transformation and collagen synthesis. In mouse models of angiotensin II-induced cardiac hypertrophy and fibrosis, knockdown of SHARPIN significantly reduced myocardial fibrosis and improved cardiac function. Conversely, higher SHARPIN expression correlated with worse fibrosis. Intriguingly, a human genetic variant of SHARPIN (rs117299156_C) was associated with lower risk of stroke in patients with myocardial infarction [33], suggesting that reduced SHARPIN activity may have protective cardiovascular effects. These findings support SHARPIN as a scaffold protein that promotes fibrogenesis by augmenting TGF-β signaling. By anchoring and prolonging the activation of Smads in cardiac fibroblasts, SHARPIN drives pathological ECM accumulation. Targeting SHARPIN or its interactions could thus be a strategy to interrupt TGF-β signaling in cardiac fibrosis.

PML—Nuclear Body Scaffold Linking TGF-β and p53 Pathways: Promyelocytic leukemia protein (PML) is a scaffold protein that forms multi-protein complexes known as PML nuclear bodies in the nucleus. PML serves as a platform for recruiting transcription factors, chromatin modifiers, and other signaling molecules. In fibrosis, PML has been shown to crosstalk with TGF-β signaling by modulating gene transcription. Specifically, PML can bind the tumor suppressor p53 and co-activate p53-dependent transcription of TGF-β target genes [54,55]. In cardiac fibroblasts, PML was found to amplify TGF-β1 responses by promoting the formation of PML-p53-Smad complexes, leading to increased expression of profibrotic genes (e.g., collagen I, α-SMA) and post-translational modifications like Smad SUMOylation that enhance signal duration. Disruption of PML (for instance, using PML inhibitors or through genetic deletion) attenuated these fibrotic responses, confirming that the PML scaffold is required for maximal TGF-β signaling in this context. PML’s role is not limited to the heart; in the liver, a long non-coding RNA (lncRNA) termed TILAM was reported to stabilize PML and form a TGF-β2-driven feed-forward loop, further boosting PML nuclear body assembly and fibrogenic gene expression [56]. Thus, PML acts as an essential nuclear scaffold that integrates TGF-β signals with other pathways (like p53) to drive fibrosis. Targeting the PML scaffold complex presents an opportunity to dial down the transcriptional output of TGF-β without directly inhibiting Smad proteins.

RACK1—Multifunctional Scaffold Regulating Smad3 Stability: RACK1 (Receptor for Activated C-Kinase 1) is a cytosolic scaffold protein with seven WD40-repeat domains, enabling it to bind a variety of signaling proteins. It is best known for anchoring protein kinase C (PKC) and membrane receptors, but in fibrosis RACK1 has a specific role in modulating TGF-β/Smad3 signaling. RACK1 binds directly to Smad3 and also interacts with ubiquitin ligases such as SPOP. By competing with SPOP for binding to Smad3, RACK1 prevents SPOP-mediated ubiquitination and degradation of Smad3. This stabilizing effect would be expected to enhance Smad3 levels and activity. Interestingly, studies in cardiac fibroblasts show a somewhat paradoxical outcome: when RACK1 is degraded, Smad3 activity is exacerbated along with increased fibrotic responses, whereas restoring RACK1 levels attenuates Smad3 activity [57]. One interpretation is that RACK1 may also scaffold inhibitory molecules or restrict Smad3 localization, so its net effect is to fine-tune Smad3 signaling rather than simply increase it. In any case, RACK1 clearly influences the intensity of TGF-β/Smad signaling in fibrosis through its scaffold function. Loss of RACK1 removes an important regulatory “docking platform” in the pathway, leading to dysregulated Smad3 activation and heightened fibrosis. Given RACK1’s involvement in many signaling pathways (TGF-β, PKC, integrins, etc.), its activity must be carefully modulated if considered for therapeutic targeting.

The TGF-β pathway section illustrates how diverse scaffold proteins—from cell membrane integrins to nuclear co-factors—orchestrate the fibrogenic TGF-β cascade. Next, we examine scaffold proteins in inflammatory and immune signaling, which often intersect with TGF-β in fibrosis.

### 4.2. Inflammatory and Immune Pathways

Pathway Background: Chronic inflammation is a well-known driver of fibrosis. In injured tissues, activation of innate immune receptors (like Toll-like receptors, IL-1 receptors) and cytokine networks (TNF-α, IL-6, interleukins) leads to the production of profibrotic mediators, recruitment of immune cells, and direct activation of fibroblasts. Key signaling pathways in inflammation—such as NF-κB and AP-1 transcription factor activation, inflammasome assembly, and JAK/STAT cytokine signaling—contribute to creating a sustained profibrotic microenvironment. Scaffold proteins are heavily involved in these pathways, as many innate immune signals rely on multi-protein complexes. Adaptor proteins like MyD88 and TRIF nucleate signaling complexes at receptors, while inflammasomes (e.g., NLRP3 inflammasome) require oligomeric scaffolds (ASC) to activate caspases, and the NF-κB pathway uses signalosomes like the CARMA/Bcl10/MALT1 (CBM) complex. In inflammatory cell-fibroblast interactions, scaffold proteins can also mediate crosstalk; for example, by controlling the release of extracellular vesicles or cytokines that affect fibroblasts. Overall, chronic inflammation and fibrosis are interlinked processes, often described as “a wound that never heals”. Targeting scaffold proteins in inflammatory pathways offers the chance to dampen both inflammation and fibrogenesis simultaneously. Below we discuss key scaffolds in innate immune signaling, NF-κB activation, and related immune pathways that have been implicated in fibrosis.

MyD88 and IRAK Signalosomes—Innate Immune Adaptor Scaffolds: MyD88 is a classic adaptor (scaffold) protein used by the Toll-like receptor (TLR) and IL-1 receptor family. Upon receptor activation by pathogen signals or damage-associated molecular patterns, MyD88 oligomerizes with IRAK kinases to form a Myddosome complex, which then activates downstream NF-κB and MAPK pathways. In fibrosis, TLR-mediated inflammation can accelerate tissue damage and scarring. For example, in Alport syndrome (a genetic kidney disease that leads to chronic inflammation and fibrosis), abnormal collagen-IV triggers TLR/MyD88 signaling in renal cells. MyD88 was shown to drive fibrosis in this model via MyD88–p38 MAPK signaling: pathologic activation led to elevated IL-1β and IL-18, enhanced NF-κB activity, and upregulation of fibrotic markers in the kidney. Treatment with tauroursodeoxycholic acid (TUDCA), a chemical chaperone that suppresses ER stress and MyD88 signaling, reduced the production of pro-inflammatory cytokines and attenuated renal fibrosis [58]. This indicates that the MyD88 scaffold is a pivotal link between immune injury signals and fibrogenesis, which is also implicated in cardiac, lung, and liver fibrosis [8,9,59]. Collectively, targeting innate immune scaffold adapters like MyD88 (e.g., with inhibitory peptides or small molecules) can interrupt the inflammatory cascade upstream, potentially preventing downstream fibrotic remodeling.

Bcl10—Scaffold of the CBM Complex in NF-κB Signaling: One of the central pathways connecting inflammation to fibrosis is NF-κB, a transcription factor that induces numerous pro-inflammatory and profibrotic genes (IL-1β, TNF-α, CCL2, etc.). Bcl10 is a crucial scaffold protein in the CARMA–Bcl10–MALT1 (CBM) signalosome complex that activates NF-κB [10]. Upon stimulation by certain receptors (e.g., antigen receptors in lymphocytes, or possibly GPCRs and TLR4 in fibrotic contexts), CARMA proteins recruit Bcl10 and MALT1, leading to IKK complex activation and NF-κB nuclear translocation. In fibrosis, Bcl10 has been studied in the context of Angiotensin II (Ang II)-induced inflammation. In a renal fibrosis model driven by Ang II, Bcl10-deficient mice showed significantly less kidney fibrosis and inflammatory cell infiltration than wild-type, indicating that Bcl10 normally promotes NF-κB–mediated inflammation and fibrogenesis [11]. Interestingly, the same Bcl10-deficient mice developed more severe proteinuria and podocyte injury, suggesting Bcl10 also has a cell-protective role in maintaining glomerular podocyte integrity. In cardiac fibrosis, Bcl10 deletion likewise attenuated Ang II-induced cardiac hypertrophy and fibrosis. These findings present Bcl10 as a double-edged sword: as a scaffold in immune cells (macrophages, etc.), Bcl10 drives inflammatory signaling that fuels fibrosis, whereas in certain structural cells, it may have additional roles. Nonetheless, Bcl10’s function as the linchpin scaffold of the NF-κB signalosome makes it an attractive target for dampening inflammation-driven fibrosis. Disrupting the Bcl10–MALT1 interaction or Bcl10 oligomerization could “turn off” a major profibrotic cytokine production pathway.

Pellino-1 (Peli1)—Ubiquitin Ligase and Signaling Scaffold: Peli1 is an E3 ubiquitin ligase that also serves as an adaptor in certain immune signaling pathways. It has emerged as a pathogenic scaffold in cardiac fibrosis under chronic pressure overload. In a mouse model of hypertensive heart disease, Peli1 was upregulated in cardiomyocytes subjected to mechanical stretch, where it facilitated crosstalk between cardiomyocytes and cardiac fibroblasts [7]. Mechanistically, Peli1 acts as a scaffold that brings together components needed for NF-κB and AP-1 activation: it promotes formation of K63-linked polyubiquitin chains that activate TAK1 and IKK, leading to NF-κB p65 and c-Jun/AP-1 signaling. This induces the production and secretion of miR-494-3p in cardiomyocytes, which are then packaged into exosomes. The Peli1-driven exosomes are taken up by neighboring fibroblasts, where miR-494-3p suppresses PTEN and consequently enhances PI3K/AKT, Smad2/3, and ERK signaling, all of which drive fibroblast activation and fibrosis. Additionally, Peli1 was found to interact with the inflammasome adaptor ASC, amplifying the activation of inflammasomes such as NLRP3. This broad action firmly establishes Peli1 as a profibrotic scaffold connecting mechanical stress to inflammatory signaling and fibroblast response. Mice lacking Peli1 were protected from cardiac fibrosis under pressure overload, showing significantly less collagen accumulation. Given these findings, Peli1 is regarded as a promising therapeutic target for cardiac fibrosis and possibly other fibrotic conditions where mechanical stress and inflammation intersect. Targeting Peli1 could simultaneously blunt NF-κB-driven inflammation and the profibrotic paracrine signaling loop between parenchymal cells and fibroblasts.

NLRP3 Inflammasome and Galectin-3—Scaffolds in Inflammasome Activation: Inflammasomes are cytosolic multi-protein complexes that sense danger signals and activate caspase-1, leading to IL-1β/IL-18 maturation and an inflammatory cascade. The NLRP3 inflammasome is strongly linked to fibrosis, as IL-1β and IL-18 are fibrogenic cytokines. NLRP3 itself oligomerizes upon activation and, together with the adaptor protein ASC, forms a large helical scaffold that recruits pro-caspase-1. While ASC is the dedicated scaffold of inflammasomes, other proteins can modulate this assembly. Galectin-3 (Gal-3) is a β-galactoside-binding lectin that can act as a pro-inflammatory scaffold. Gal-3 has a tendency to oligomerize and crosslink glycosylated proteins on cell surfaces or in the extracellular space. In the context of inflammasomes, Gal-3 was shown to enhance NLRP3 inflammasome activation and IL-1β production. In a model of IgA nephropathy with renal fibrosis, Gal-3 deficiency led to reduced NLRP3 activation, lower IL-17 and IL-1β levels, and attenuation of fibrosis, whereas Gal-3 was found to promote T helper 17 (Th17) cell differentiation [12]. By binding to receptors or ECM components, Gal-3 can cluster cell-surface signaling molecules and facilitate the formation of the NLRP3 inflammasome complex, effectively acting as an extracellular scaffold that augments innate inflammatory signaling. Inhibitors of Gal-3 (such as modified citrus pectin or novel polysaccharides) have shown antifibrotic effects by disrupting these interactions (discussed in Section 5). Thus, targeting Gal-3 breaks a critical link between chronic inflammation and fibrosis.

CUL4B—E3 Ubiquitin Ligase Scaffold in Macrophage Activation: CULLIN-4B (CUL4B) is not a traditional signaling adaptor, but as part of Cullin–RING E3 ubiquitin ligase complexes it serves a scaffold-like role assembling ubiquitination machinery. Recent studies indicate CUL4B is involved in fibrotic immune responses. Myeloid cell-specific deletion of CUL4B in mice led to reduced macrophage infiltration and fibrosis in models of tissue injury [60]. This suggests that CUL4B in macrophages might stabilize or promote the turnover of key signaling proteins that drive macrophage activation and profibrotic cytokine production. By acting as a molecular scaffold for E3 ligase components and substrates, CUL4B can regulate signaling pathways such as NF-κB or STAT3 in macrophages. The reduced fibrosis in CUL4B knockout myeloid cells highlights its significance in immune cell-mediated fibrogenesis. Targeting such ubiquitin ligase scaffolds could modulate the inflammatory cell contribution to fibrosis, although specificity would be a challenge given the broad role of Cullin complexes.

Downstream Cytokine Signaling—JAK2/STAT and SH2B3: Chronic inflammatory environments in fibrosis are rich in cytokines (IL-6, IL-11, interferons, etc.) that signal via the JAK/STAT pathway. Intriguingly, signaling components themselves can moonlight as scaffolds. Janus kinase 2 (JAK2), beyond its kinase activity, has been noted to function as a scaffold stabilizing STAT transcription factor dimers at receptor complexes. In a study of post-infarction cardiac fibrosis, the pro-inflammatory and metabolic factor PCSK9 was found to drive myofibroblast activation through JAK2/STAT3 signaling [61]. JAK2 formed a stable complex with STAT3, maintaining STAT3 phosphorylation and nuclear activity. This scaffolding role of JAK2 amplified PCSK9-induced expression of fibrogenic genes, whereas inhibition of STAT3 or JAK2 could reduce cardiac fibrosis. This indicates that interfering with the JAK2-STAT3 complex assembly (for instance with peptidomimetics) might dampen fibrosis beyond what simple kinase inhibition can achieve. Another relevant adaptor is SH2B3 (also known as Lnk), a scaffold protein in immune cells that normally attenuates cytokine signaling. A common SH2B3 polymorphism (R262W, rs3184504) impairs its ability to inhibit IL-12/STAT4 signaling. SH2B3 negatively regulates the JAK-STAT signaling pathway by inhibiting JAK2 activity. The Trp-encoding allele of rs3184504 leads to reduced SH2B3 function, enhancing the JAK2-STAT4-IFNγ signaling axis and promoting inflammation and fibrosis [62,63,64]. Therefore, JAK2 plays a critical role in the SH2B3-mediated fibrotic mechanism, and therapeutic strategies targeting JAK2 may hold potential value for related diseases.

Filamin A—Linking Inflammatory Receptors to Downstream Signaling: Filamin A (FLNA) is a large cytoskeletal scaffold that can connect cell surface receptors to intracellular signaling pathways. It crosslinks actin filaments but also has multiple binding partners, effectively serving as an anchor between the cytoskeleton and signaling proteins. In fibrotic liver disease (such as NASH-related fibrosis), FLNA is highly expressed in liver macrophages and hepatic stellate cells (HSCs) [29]. FLNA may connect TLR4 and STAT3 and promote the inflammatory response in NASH. This scaffold mechanism facilitates robust STAT3 activation in response to TLR4 stimulation, leading to production of IL-6, TNF-α, chemokines like CXCL10 in macrophages, and TGF-β1 and CCL2 in HSCs. Concurrently, FLNA in HSCs suppresses the expression of matrix-degrading MMPs while enhancing fibrogenic factors, thereby linking inflammation to reduced matrix turnover and increased collagen deposition. Knockdown of FLNA disrupted this inflammatory crosstalk: it reduced pro-inflammatory cytokine release by macrophages and lowered fibrotic marker expression in HSCs, ultimately attenuating liver fibrosis. Thus, FLNA acts as a critical scaffold connecting Toll-like receptor signaling to profibrotic transcriptional responses in both immune cells and fibroblasts. Targeting the FLNA scaffold (for example, with peptides that block its interaction with TLR4 or STAT3) could simultaneously impair the inflammatory stimulus and the fibrogenic response.

In summary, numerous scaffold proteins coordinate the inflammatory and immune signals that drive fibrosis. They operate at different levels, from initial receptor signaling (MyD88, TRAF6, Peli1), to intermediate signalosomes (Bcl10, ASC, Galectin-3), to downstream transcriptional regulation (such as JAK2/STAT complexes). By holding the signaling modules for cytokine production and immune cell activation together, these scaffolds amplify inflammation in a way that feeds into fibrosis. Conversely, some scaffolds like SH2B3 act as braking mechanisms that, when lost, exacerbate fibrotic inflammation. Therapeutically, modulating these scaffolds holds promise for damping the chronic inflammation that accompanies fibrosis. Indeed, interventions like Galectin-3 inhibitors or MyD88 pathway blockers have shown fibrosis reduction in preclinical models. However, care must be taken to preserve host defense functions. The next sections will turn to developmental and homeostatic pathways (Wnt/β-catenin) and mechanotransduction, which also rely on scaffolding mechanisms in fibrosis.

### 4.3. Wnt/β-Catenin Pathway

Pathway Background: The Wnt/β-catenin signaling pathway is essential for development and tissue regeneration, but its aberrant reactivation in adult tissues is frequently implicated in organ fibrosis. Canonical Wnt signaling is initiated when Wnt ligands bind to Frizzled/LRP receptors, leading to stabilization and nuclear accumulation of β-catenin, which then drives transcription of target genes (e.g., fibronectin, cyclin D1, MMP7). In healthy adults, Wnt signaling is usually quiescent or tightly regulated by a “β-catenin destruction complex” that targets β-catenin for degradation. In fibrotic diseases, sustained Wnt activity has been observed—for instance, in IPF lungs, in failing hearts, and in chronically injured kidneys and livers—contributing to fibroblast activation, epithelial-to-mesenchymal transition, and stem cell exhaustion. Scaffold proteins are integral to both activation and repression in the Wnt pathway. They form complexes at the membrane to propagate Wnt signals, and they assemble the cytosolic destruction complex that keeps β-catenin levels in check. Below we discuss key scaffolds such as Axin and Dishevelled that mediate Wnt signaling, as well as their links to fibrosis.

Axin1—Core Scaffold of the β-Catenin Destruction Complex: Axin1 is a quintessential scaffold protein in the canonical Wnt pathway. It serves as the central platform for the β-catenin destruction complex, binding GSK-3β (a kinase that phosphorylates β-catenin), APC (a tumor suppressor and scaffold that also binds β-catenin), and other components like CK1. By holding this complex together, Axin1 facilitates the phosphorylation and subsequent proteasomal degradation of β-catenin, thus preventing Wnt target gene activation. In fibrotic conditions, reduced Axin1 expression or function has been linked to excessive Wnt signaling. For example, in a lung fibrosis model, partial loss of Axin1 correlated with increased β-catenin accumulation and more severe fibrosis [13,14]. Conversely, restoring Axin1 levels or enhancing its stability led to greater β-catenin degradation and attenuation of Wnt-driven fibrogenesis. Axin1 is often considered a “brake” on Wnt signaling; when that brake fails (due to low Axin or active Wnt signaling which inactivates the destruction complex), β-catenin can accumulate unchecked and promote fibrotic gene programs. Axin’s importance in fibrosis is underscored by experimental interventions: strategies that upregulate Axin or mimic its scaffold function (for instance, small molecules stabilizing Axin) have shown antifibrotic effects in preclinical studies. Thus, Axin1’s scaffolding of the destruction complex is a critical control point preventing inappropriate Wnt/β-catenin activation during tissue injury.

Dishevelled (Dvl)—Membrane-Proximal Wnt Signal Scaffold: Dishevelled proteins (Dvl1, Dvl2, Dvl3) are cytoplasmic scaffolds that transduce Wnt receptor activation to downstream effects. Upon Wnt ligand binding, Dvl is recruited to the Frizzled receptor at the membrane, where Dvl polymerizes and serves as a scaffold to inhibit the Axin–GSK-3β complex. Dvl contains DIX, PDZ, and DEP domains, enabling it to bind Frizzled, Axin, and other factors, essentially forming a bridge from the receptor to the destruction complex. In fibrosis, elevated Dvl expression has been observed as a mediator of heightened Wnt signaling. For example, Dvl1 was found upregulated in fibrotic lung tissues, and its activity was modulated by a microRNA (miR-92a-2-5p) in one IPF study [13]. Inhibiting that microRNA led to increased Dvl1 and exacerbated Wnt signaling, suggesting that normally miR-92a-2-5p kept Dvl1 in check to restrain fibrosis. These findings implicate Dvl1 as a profibrotic Wnt scaffold whose level is fine-tuned by non-coding RNAs. However, direct evidence linking Dvl proteins to fibrogenesis (such as Dvl knockouts in fibrosis models) remains limited. No fibrotic phenotype has been definitively attributed to Dvl loss or overexpression yet, likely due to redundancy among Dvl isoforms and the complexity of Wnt signaling. It is suspected that Dvl-mediated Wnt amplification contributes to fibrosis, but further investigation is needed. Regardless, Dvl is an attractive target to consider; small molecules that disrupt Dvl polymerization (and thus its scaffold function) could prevent the propagation of Wnt signals in fibrotic tissues.

Numb—Adaptor Modulating Wnt and Notch Signaling: Numb is an endocytic adaptor protein traditionally known for cell fate determination (antagonizing Notch signaling by targeting Notch receptors for degradation). It is not a canonical component of Wnt pathways, but Numb can influence Wnt/β-catenin signaling and has been linked to fibrosis. In alveolar epithelial cells, Numb overexpression was shown to activate Wnt/β-catenin signaling, driving transdifferentiation processes associated with pulmonary fibrosis. The mechanism may involve Numb stabilizing β-catenin or affecting the turnover of Wnt receptors, although details are still being uncovered. Notably, Numb’s role appears cell-type specific: in the lung epithelium it amplifies Wnt signals (promoting a more mesenchymal, fibrotic phenotype), whereas in other contexts (like the kidney or liver) Numb primarily interacts with the autophagy and Notch pathways. The dual functionality of Numb—acting as a scaffold/adaptor for different signaling molecules—highlights how one scaffold protein can have divergent effects on fibrosis depending on the cellular environment. In pulmonary fibrosis, Numb’s enhancement of Wnt signaling may contribute to aberrant epithelial regeneration and fibroblast activation [65]. In summary, while Numb is not a classical Wnt pathway scaffold, its ability to modulate Wnt/β-catenin activity places it among the factors linking Wnt signaling to fibrotic outcomes. Importantly, strategies to manipulate Numb (for instance, altering its expression via cell therapy as noted later) could shift the balance of Wnt and Notch signals in ways beneficial for tissue repair over fibrosis.

Crosstalk: The Wnt/β-catenin pathway does not act in isolation. It intersects with TGF-β and other pathways, often via shared scaffold proteins or induced feedback loops. For example, TGF-β can upregulate Wnt ligands and Dvl expression, while β-catenin/TCF can induce TGF-β and ECM genes. Scaffolds like IQGAP1, discussed earlier, can bind components of both Wnt (β-catenin) and TGF-β (Smads, indirectly via actin) pathways, coordinating their activities. Additionally, Axin interacts with components of the JNK pathway and has roles in regulating SMAD3 in some contexts, illustrating that scaffold proteins can integrate multiple signaling inputs. Understanding these connections is vital, as combination therapies might be needed to disrupt profibrotic signaling networks effectively.

In summary, the Wnt/β-catenin pathway relies on scaffolding at multiple steps; Axin holding together the inhibitory complex and Dishevelled transmitting activation at the membrane. Dysregulation of these scaffolds tends to favor fibrogenesis by unleashing β-catenin-driven transcription. Therapeutically, bolstering the function of negative scaffolds (like Axin1) or inhibiting positive scaffolds (like Dvl) could help restore control over Wnt signaling in fibrotic diseases. Approaches such as stabilization of Axin (there are small molecules called “tankyrase inhibitors” that prevent Axin degradation) are being explored in fibrotic models as a way to promote β-catenin destruction. The next section will address mechanotransduction pathways, which often act upstream of TGF-β and Wnt, converting physical matrix signals into biochemical cascades during fibrosis.

### 4.4. Mechanotransduction Pathways

Pathway Background: Mechanotransduction refers to the processes by which cells sense and respond to mechanical forces. In fibrosis, developing scar tissue alters the mechanical properties of the tissue (increased stiffness, altered shear forces), which in turn feeds back to cells to further modulate their behavior. Fibroblasts, for example, can sense a stiffened matrix and respond by differentiating into myofibroblasts and producing even more collagen, a feed-forward loop driving progressive fibrosis. Key signaling pathways involved in mechanotransduction include focal adhesion signaling, Rho/ROCK-mediated cytoskeletal tension, YAP/TAZ activation via the Hippo pathway, and stretch-activated ion channel signaling. Scaffold proteins are central to mechanotransduction, as the sensing of mechanical cues often involves large multi-protein complexes at the cell–ECM interface (focal adhesions, integrin complexes, ion channel complexes). By linking external forces to internal signaling cascades, these scaffolds effectively translate a biophysical stimulus into a biochemical signal that can alter gene expression. In fibrotic diseases, mechanotransduction scaffolds become particularly important because the ECM is abnormally stiff; thus, mechanosignaling pathways may be constantly activated. Below we highlight several scaffolds that mediate mechanical signaling in fibrosis, including integrins and caveolin-1 at the membrane, and specialized scaffolds that enable invasive cell behavior under mechanical stress.

Integrins and Caveolin-1—Membrane-ECM Linker Scaffolds: Integrins (discussed earlier in the TGF-β section) are prime mechanotransducers. They cluster into focal adhesions, recruiting scaffolding proteins like talin, paxillin, vinculin, and focal adhesion kinase (FAK) to form a signaling hub. In fibrosis, integrin-mediated adhesion to a stiff ECM leads to activation of FAK and downstream pathways such as TGF-β, PI3K/AKT, and YAP/TAZ. We have already noted the role of α6β1 integrin as a mechanosensitive scaffold in lung fibrosis that connects matrix stiffness to TGF-β activation. Many other integrins contribute similarly; for instance, αv integrins activate latent TGF-β by mechanical force, and β1 integrins convey tensile signals to the nucleus via the LINC complex. Transglutaminase 2 (TG2) plays a central role in ECM remodeling by catalyzing irreversible crosslinking of ECM proteins like collagen and fibronectin, driving fibrosis progression. Monoclonal antibodies targeting TG2’s catalytic core have shown nanomolar efficacy in inhibiting transamidase activity and ECM deposition in fibrosis models [66,67]. In parallel, TG2 inhibitors, such as LDN 27219, provide additional antifibrotic strategies [68]. Lysyl oxidase-like protein 2 (LOXL2), another ECM regulator, facilitates collagen crosslinking and fibrosis [69]. Its regulation by NUDT21 and therapeutic targeting through siRNA-loaded liposomes have significantly reduced fibrosis in silicosis models, highlighting its potential for antifibrotic interventions [70]. Caveolin-1 (Cav1) is a critical membrane scaffold involved in mechanosensing. Cav1 is the principal structural protein of caveolae, flask-shaped invaginations of the plasma membrane that buffer membrane tension. Cav1 has a scaffolding domain that binds signaling molecules (e.g., eNOS, Src family kinases, TGF-β receptors), organizing them within caveolae. In fibrotic tissues, mechanical stress and stretch can cause caveolae to flatten, releasing Cav1-bound molecules and initiating signaling cascades. In liver fibrosis, for example, increased matrix stiffness leads to enhanced Cav1-mediated endocytosis and vesicular trafficking in hepatic stellate cells [15]. Cav1 scaffolds a complex involving the tissue inhibitor of metalloproteinases TIMP-1, promoting its secretion in exosomes and thereby reducing ECM degradation. Cav1 knockdown in these cells reduced the aberrant TIMP-1 secretion and ameliorated fibrosis in experimental models. Cav1 deficiency exacerbates inflammation and fibrosis in pressure overload models, whereas Cav1 overexpression is protective [15,16,17]. These context-dependent effects highlight that Cav1 is a mechanotransduction scaffold whose impact varies; it can either restrain fibrosis by organizing protective signals or, if dysregulated, mediate harmful signals. Overall, integrins and Cav1 represent the cell’s primary interface with the mechanical microenvironment. By assembling focal adhesion and caveolar complexes, they convert external matrix stiffness and tension into intracellular signals that control fibroblast activation, proliferation, and survival.

TKS5—Scaffold for Invasive Podosomes and ECM Degradation: As fibrosis progresses, activated myofibroblasts often migrate and invade through tissues, contributing to scar expansion and disrupting normal tissue structure. This invasive behavior is driven by mechanical cues and requires specialized structures called podosomes or invadopodia, actin-rich protrusions that concentrate matrix-degrading enzymes. TKS5 (SH3PXD2A) is a scaffold protein that is critical for forming and organizing podosomes. TKS5 contains multiple SH3 domains and a PX domain, allowing it to localize to membrane protrusions and recruit MMPs and actin regulators. In fibrotic tissues, TKS5 is upregulated in myofibroblasts experiencing high mechanical stress. TKS5 serves as an anchor for matrix metalloproteinases (MMP-2, MMP-9, MMP-14) at invasive fronts, facilitating focused ECM proteolysis. In pulmonary fibrosis models, elevated TKS5 expression was shown to promote fibroblast invasion through basement membranes and interstitial matrix [18,19]. Conversely, pharmacological inhibition or knockdown of TKS5-impaired podosome formation, reduced pericellular collagen degradation, and attenuated the invasiveness of fibroblasts, ultimately leading to decreased pathological matrix deposition. Thus, TKS5 acts as a mechano-responsive scaffold that endows fibroblasts with invasive capacity. By assembling the proteinases and structural proteins needed to breach matrix barriers, TKS5 links mechanical stimulation (stiff matrix, TGF-β-induced motility) to the tissue remodeling characteristic of aggressive fibrosis. Targeting TKS5 or its interacting partners could help contain fibrosis by limiting the ability of myofibroblasts to spread and destroy healthy tissue architecture.

TRPV4–PI3Kγ Complex—Mechanosensing Ion Channel Scaffold: TRPV4 is a calcium-permeable mechanosensitive ion channel expressed in many cell types, including fibroblasts. Under increased matrix stiffness or cyclic stretch, TRPV4 channels open and allow Ca^2+^ influx, which can trigger various downstream signals. A novel finding in lung fibrosis is that TRPV4 physically associates with PI3Kγ, a class I phosphoinositide 3-kinase isoform, forming a mechanosensitive signaling module. Upon mechanical stimulation of lung fibroblasts on a stiff matrix, activated TRPV4 channels were found to recruit and cluster PI3Kγ at the membrane. This proximity leads to localized production of PI3Kγ and activation of AKT signaling [27]. The TRPV4–PI3Kγ complex thus acts as a scaffolded signaling unit converting a mechanical cue (stretch or stiffness sensed by TRPV4) into a biochemical response (AKT pathway activation). The outcome is promotion of fibroblast differentiation into myofibroblasts and excessive ECM production [27,28]. Inhibiting either component of this complex—with a TRPV4 channel blocker or a PI3Kγ inhibitor—was sufficient to disrupt the mechano-activated AKT signaling and mitigate lung fibrogenesis in stiffness-induced fibrosis models. This example highlights how even a membrane channel and an enzyme can function together as a scaffolded complex: TRPV4’s large cytosolic domains likely serve as a docking site for PI3Kγ and possibly other signaling proteins. Interrupting the assembly of this mechanotransduction complex offers a targeted way to stop mechanical signal amplification in fibrosis.

In fibrotic microenvironments, cells are subjected to abnormal mechanical forces, and scaffold proteins at the cell periphery and inside the cell ensure these forces are translated into cellular responses. Key mechanotransduction scaffolds can be categorized as: (1) signal transducers, such as integrins and Cav1, which convert external mechanical stimuli into activation of canonical signaling pathways like TGF-β or NF-κB; and (2) effector executors, such as TKS5 and TRPV4–PI3Kγ, which confer cells with the machinery to remodel the ECM and generate force. These scaffolds have well-documented roles across multiple organ fibroses: for instance, α6β1 integrin mediates mechanosignaling in lung fibrosis, Cav1 links membrane tension to fibrogenic secretion in liver fibrosis, TKS5 drives matrix degradation in lung fibrosis, and TRPV4–PI3Kγ triggers myofibroblast differentiation in stiff lung tissue. By targeting such scaffolds, one can aim to “decouple” mechanical stress from the biochemical fibrotic cascade, essentially breaking the self-perpetuating loop of matrix stiffening leading to more fibrosis. Approaches might include integrin antagonists (several are in trials for fibrosis), caveolin-mimetic peptides to restore caveolae function, or inhibitors of mechanosensitive channels. Another interesting scaffold in mechanotransduction is STAP2 (Signal Transducing Adaptor Family Member 2), which was identified in kidney fibrosis: STAP2 was found to scaffold the heat-shock protein HSP27 to PI3K/AKT signaling complexes upon integrin engagement, thereby promoting collagen synthesis and epithelial–mesenchymal transition [71]. This again underscores the theme that scaffolds often tie mechanical signals to classic profibrotic pathways like PI3K/AKT. In the future, mapping the interactome of scaffold proteins under mechanical strain conditions will further clarify how mechanical and chemical signals converge in fibrosis.

### 4.5. Other Fibrosis-Related Pathways

Beyond TGF-β, immune, Wnt, and mechanical signaling, a variety of other pathways contribute to fibrosis, often in cell-type or context-specific ways. These include cyclic AMP (cAMP)/PKA signaling, PI3K/AKT metabolic signaling, oxidative stress responses (e.g., Nrf2 pathway), autophagy and mitophagy pathways, and epigenetic/transcriptional regulation. Recently, a review suggests that the PTHrP (Parathyroid Hormone-Related Protein) pathway can link the TGF-β1, PKA/cAMP, and Akt/Foxo signaling pathways [72]. Scaffold proteins also play prominent roles in these areas, frequently serving as points of integration between the major pathways discussed above. For instance, a scaffold protein might connect a growth factor signal to a metabolic pathway, or link inflammatory signals to autophagy. We highlight a few representative scaffold proteins in these categories that have been implicated in fibrotic diseases, emphasizing how they qualify as scaffolds and what evidence ties them to fibrosis.

cAMP/PKA Pathway—AKAPs in Fibrosis: The cyclic AMP/PKA signaling pathway usually has antifibrotic effects (promoting relaxation, antiproliferative signals), and A-Kinase anchoring proteins (AKAPs) are the prototypical scaffolds for this pathway. AKAPs tether PKA and other enzymes to specific subcellular locations, thereby compartmentalizing cAMP signaling. Among the large AKAP family, AKAP2 has been studied in cardiac remodeling and fibrosis. AKAP2 is a multivalent scaffold that can anchor PKA as well as other signaling molecules. Intriguingly, AKAP2 plays dual roles in the heart depending on the cell type: in cardiomyocytes, AKAP2 scaffolds PKA and the transcriptional coactivator SRC-3, which leads to activation of estrogen receptor α and increased expression of pro-survival and pro-angiogenic genes (like BCL-2 and VEGFA); this is beneficial, helping cardiomyocytes resist apoptosis and indirectly reducing fibrosis after myocardial infarction [23]. On the other hand, in cardiac fibroblasts (myofibroblasts), AKAP2 localizes to F-actin stress fibers and assembles a profibrotic signaling complex: it brings together the adaptor Grb2, the kinase ERK1, and the actin regulator WAVE2. This AKAP2/ERK1/WAVE2 complex drives actin polymerization and cell migration, thereby promoting the invasive, contractile behavior of myofibroblasts [23,24]. Silencing AKAP2 or disrupting its interaction with actin impairs ERK1 activation of WAVE2 and significantly reduces fibroblast migratory capacity, translating to attenuated cardiac fibrosis and decreased tissue stiffness. Thus, AKAP2 exemplifies how a scaffold protein can integrate cAMP/PKA signaling with growth factor pathways (ERK) and cytoskeletal dynamics. By doing so, it influences both the survival of parenchymal cells and the activity of fibrogenic cells. AKAP2’s scaffolding function is central to its effects; it would not coordinate these disparate signals without its multi-domain structure capable of binding PKA, Src/Src-like molecules, and actin. Given its pivotal position, AKAP2 is being considered as a therapeutic target for cardiac fibrosis, with the challenge being to inhibit its profibrotic scaffold interactions in fibroblasts while perhaps sparing or even enhancing its cardioprotective scaffold role in cardiomyocytes.

Another member, AKAP12 (gravin), primarily exerts antifibrotic effects in certain contexts. AKAP12 is a scaffold that interacts with PKA and PKC and localizes to the cell membrane. In the liver, hepatocyte-specific deletion of AKAP12 was found to exacerbate fibrosis: loss of AKAP12 led to overactivation of the PI3K/AKT pathway (due to increased activity of a protease PCSK6 that was normally kept in check) [30]. The result was heightened macrophage infiltration, oxidative stress, and inflammatory cytokine release, all of which worsened liver injury and fibrosis. Knocking down PCSK6 partially rescued this phenotype, indicating that AKAP12’s scaffold function includes restraining PI3K/AKT signaling via modulation of upstream factors like PCSK6. Additionally, AKAP12 can scaffold components that inhibit inflammatory signaling (it has been reported to sequester or regulate PKA phosphorylation of targets that affect STAT3 and NF-κB) [31,32]. As chronic liver disease progresses, AKAP12 levels tend to decline, removing its restraint on profibrotic pathways, whereas interestingly during recovery from fibrosis, AKAP12 levels rise again. In hepatic stellate cells, phosphorylation of AKAP12 was found to disrupt its binding to the collagen chaperone HSP47, leading to excessive collagen secretion and ER stress. Restoration of AKAP12’s normal function (for example, via gene editing to prevent that phosphorylation) alleviated fibrosis in experimental systems. These findings paint AKAP12 as a scaffold that normally maintains homeostasis by anchoring signaling proteins that suppress fibrogenic pathways. When AKAP12 is lost or inactivated, unchecked AKT, STAT3, and NF-κB signaling drive fibrosis. Thus, AKAP12 stands in contrast to many profibrotic scaffolds; it is more of a protective scaffold, at least in the liver and kidney context. Supporting such scaffolds or mimicking their interactions could be a therapeutic angle to bolster antifibrotic defenses.

Oxidative Stress Pathway—CKIP-1 (Casein Kinase-2 Interacting Protein): Oxidative stress is often both a cause and consequence of fibrosis. Reactive oxygen species (ROS) can activate TGF-β, damage cells, and alter gene expression, while fibrotic cells often produce excess ROS through NADPH oxidases or mitochondrial dysfunction. The Nrf2 pathway is the master regulator of antioxidant responses; when activated, Nrf2 induces genes that counteract oxidative stress. CKIP-1 is a scaffold protein that has emerged as a positive regulator of the Nrf2 pathway in fibrotic settings. CKIP-1 (also known as PLEKHO1) interacts with casein kinase 2 and other proteins, functioning as an adaptor in multiple signaling contexts. In the kidney, studies of diabetic kidney disease (which involves oxidative stress and fibrosis) showed that CKIP-1 is required for effective Nrf2 activation. Specifically, the C-terminal domain of connexin 43 (a gap junction protein, Cx43) can bind CKIP-1; this interaction stabilizes CKIP-1 and facilitates Nrf2 nuclear translocation [73]. Under high glucose (diabetic) conditions, Cx43 is downregulated or mislocalized, weakening the Cx43–CKIP-1 interaction and thus destabilizing CKIP-1. Furthermore, Src kinase can phosphorylate CKIP-1 and promote its ubiquitination and degradation [74]. The loss of CKIP-1 leads to impaired Nrf2 signaling, insufficient induction of antioxidant genes, and accumulation of oxidative damage, which in turn exacerbates renal fibrosis. In experimental models, overexpressing CKIP-1 or preventing its degradation enhanced Nrf2 activity, reduced oxidative stress, and blunted fibrogenesis. Therefore, CKIP-1 serves as a scaffold for the antioxidant defense machinery: by anchoring and assisting Nrf2 (possibly by bringing Nrf2 and its import mechanisms together), CKIP-1 helps cells adapt to oxidative stress. Therapies that stabilize CKIP-1 or mimic its effect (for example, using peptides that enhance the Cx43–CKIP-1 interaction) could amplify the cell’s own antifibrotic, antioxidant responses. Conversely, in fibrotic diseases where CKIP-1 is often downregulated, its loss is a contributing factor to the persistent oxidative milieu.

Another example in the oxidative stress category is PDZK1, a scaffold that links membrane transporters to signaling pathways. PDZK1 has PDZ domains that bind to various transport proteins, including the carnitine/organic cation transporter OCTN1. In the kidney, TGF-β1 was found to strongly downregulate PDZK1, leading to reduced OCTN1 function and diminished uptake of the dietary antioxidant ergothioneine [34]. The loss of ergothioneine and other antioxidants due to low PDZK1 levels increases cellular oxidative stress and aggravates fibrotic signaling. Additionally, PDZK1 can interact with signaling molecules in the cytosol; it has been reported to modulate the PI3K/AKT and p38 MAPK pathways, which in turn affect oxidative stress responses and epithelial–mesenchymal transition. Overexpression of PDZK1 in a mouse model counteracted TGF-β’s profibrotic effects by boosting antioxidant defenses (through enhanced ergothioneine uptake) and reducing markers of EMT and fibrosis. Thus, PDZK1 acts as a scaffold connecting extracellular nutrient signals (antioxidants) with intracellular survival pathways, ultimately protecting against fibrosis. This highlights how metabolic and redox aspects of fibrosis can be governed by scaffold proteins that one might not traditionally think of as signaling hubs.

Autophagy and Mitophagy—NDP52 and SPAG9 (JLP): Autophagy is the process of cellular self-digestion that removes damaged organelles and protein aggregates. In fibrosis, autophagy plays a double-edged role: mild autophagy can be protective by clearing ROS-producing mitochondria and limiting inflammasome activation, but some studies suggest certain autophagy pathways in myofibroblasts might actually support their survival. Scaffold proteins regulate selective forms of autophagy such as mitophagy (mitochondrial autophagy). NDP52 (CALCOCO2) is a selective autophagy receptor that acts as a scaffold to link cargo (like damaged mitochondria) to the autophagic machinery. It has domains that bind ubiquitin on damaged organelles and LC3 on autophagosomes, thereby bridging the cargo to the autophagosome membrane. In the context of myocardial infarction-induced cardiac fibrosis, NDP52 was shown to be protective. After cardiac injury, NDP52 promotes mitophagy; it helps recruit the kinase TBK1 and the late endosome protein RAB7 to autophagosomes, facilitating the fusion of autophagosomes with lysosomes and the efficient clearance of damaged mitochondria. If NDP52 is absent, dysfunctional mitochondria accumulate in cardiomyocytes, leading to excess ROS production, which in turn increases cardiomyocyte death and triggers inflammation and fibrosis in the heart [75]. Thus, NDP52 functions as a scaffold organizing the mitophagy machinery to prevent post-injury fibrosis. Enhancing NDP52 activity (or using drugs that activate mitophagy) could be a strategy to limit oxidative damage and subsequent fibrotic remodeling following myocardial infarction.

Another scaffold linking autophagy and fibrotic signaling is SPAG9 (JLP). SPAG9, also known as JNK-associated Leucine Zipper protein (JLP), is a scaffold for MAPK signaling; it can bind JNK and p38 MAPK modules. In kidney fibrosis, SPAG9 was found to have an antifibrotic role by promoting autophagy. Overexpression of SPAG9 in tubular epithelial cells inhibited TGF-β1-induced epithelial–mesenchymal transition and collagen production [35]. Mechanistically, SPAG9 scaffolded a complex that includes Beclin-1 (a key autophagy initiator) and thus enhanced autophagy flux, which led to suppression of TGF-β/Smad3 signaling. In vivo, SPAG9 overexpression or stimulation mitigated renal fibrosis, whereas low SPAG9 levels correlated with more severe fibrosis. This indicates that the SPAG9 scaffold integrates stress signaling (p38/JNK) with autophagic pathways to counter fibrosis. It might be that SPAG9, by bringing together MAPK and autophagy components, triggers a cell stress response that degrades profibrotic signaling proteins or improves cellular housekeeping, thereby antagonizing fibrogenesis. SPAG9’s example is insightful because it shows a scaffold protein that is traditionally part of a pro-inflammatory kinase pathway can actually protect against fibrosis when it diverts signaling towards autophagy.

Epigenetic and Transcriptional Regulation—BRD4: Fibrosis involves not just acute signaling changes but also longer-term reprogramming of gene expression in cells (often termed a fibrotic “phenotype” switch). Epigenetic readers and chromatin scaffolders like BRD4 (bromodomain-containing protein 4) have emerged as central to sustaining fibrotic gene expression. BRD4 binds to acetylated histones and gathers transcriptional machinery at super-enhancers, large regulatory DNA regions that drive high expression of genes, many of which are involved in fibrosis (collagens, cytokines, etc.). In this sense, BRD4 is a chromatin scaffold: it tethers together acetylated nucleosomes, transcription factors, and co-activators such as p300/CBP to form an active transcriptional hub [20,21,22]. In hepatic stellate cells, for instance, TGF-β1 signaling leads to establishment of super-enhancers at fibrosis-related genes, and BRD4 is required to maintain these, promoting persistent collagen I and α-SMA expression. Knockdown of BRD4 in HSCs attenuated their activation and ECM production, and treatment of fibrotic mice with a BRD4 inhibitor (the BET bromodomain inhibitor JQ1) significantly reduced liver fibrosis. BRD4 also mediates crosstalk between inflammatory signals and fibrotic genes: in macrophages and cardiac fibroblasts, BRD4 was shown to form complexes with transcription factors IRF3 and NF-κB p65, driving the expression of pro-inflammatory and profibrotic genes [76,77]. In a cardiac injury model, macrophage-specific deletion of BRD4 reduced IL-1β and TNF-α levels and lessened cardiac fibrosis, highlighting BRD4’s role in linking inflammation to fibrosis. Furthermore, BRD4 can repress antifibrotic factors; in alcoholic liver disease, BRD4 binding to the SIRT1 promoter suppresses SIRT1 expression, which leads to impaired autophagy and heightened TGF-β signaling [78]. All told, BRD4 acts as a scaffolding platform in the nucleus that sustains the fibrogenic transcriptional program. It connects epigenetic marks (histone acetylation) to sustained gene transcription and integrates multiple pathways (TGF-β, NF-κB, etc.) at the chromatin level. This makes BRD4 an especially attractive therapeutic target; inhibiting BRD4 can “unravel” the profibrotic transcriptional complexes and allow cells to revert from the activated myofibroblast state. Indeed, several BRD4 inhibitors are in development or early trials for fibrotic diseases. As we will see in the next section, some of the most promising experimental therapies for fibrosis involve targeting scaffold proteins like BRD4 and others we have discussed.

## 5. Therapeutic Targeting of Scaffold Proteins in Fibrosis

Given their central role in orchestrating fibrogenic signaling, scaffold proteins represent enticing targets for antifibrotic therapy. However, targeting scaffold proteins is challenging because they lack enzymatic activity and often participate in multiple pathways. Despite this, recent research has identified several strategies to modulate scaffold proteins for therapeutic benefit. These include small-molecule inhibitors, peptides that disrupt protein–protein interactions, biologics (like engineered antibodies or decoy receptors), gene therapy approaches, and even cell-based therapies. Here we highlight notable therapeutic approaches from preclinical (and in some cases translational) studies where scaffold proteins in fibrosis were directly targeted. We focus only on interventions that go beyond genetic knockdown; i.e., actual therapeutic agents or delivery methods tested to modulate scaffold function and ameliorate fibrosis (Table 3).

Targeting TGF-β Pathway Scaffolds: One successful strategy has been to interfere with scaffold-mediated TGF-β signaling. For example, integrin α6β1—a key mechanotransduction scaffold for TGF-β activation (Section 4.1)—was targeted in lung fibrosis models. Treatment with a functional blocking antibody against α6 integrin or small-molecule inhibitor significantly reduced pulmonary fibrosis in mice, decreasing fibroblast invasion and collagen deposition [1]. This validates integrins as druggable scaffolds that link mechanical cues to TGF-β; indeed, αvβ6 integrin inhibitors (like the antibody STX-100) have entered clinical trials for IPF but failed. Another approach in the TGF-β pathway is targeting caveolin-1 (Cav1). A caveolin-1-derived peptide called CSP7 (Cav1 scaffolding domain peptide) was developed to disrupt pathological Cav1 interactions [79]. In a murine pulmonary fibrosis model, CSP7 administration inhibited TGF-β/Smad3 signaling and restored autophagy in alveolar epithelial cells, leading to reduced collagen accumulation and fibrosis [80]. CSP7 essentially mimics the Cav1 scaffold domain to sequester and inhibit the TGF-β receptor–Smad complex, demonstrating the feasibility of using peptide therapeutics against scaffold protein interactions. Downstream in the nucleus, BRD4 has been a high-profile target: the small-molecule BET bromodomain inhibitor JQ1 prevents BRD4 from binding acetylated chromatin, thereby dismantling the fibrotic super-enhancer complexes. JQ1 and related inhibitors have shown efficacy in reducing fibrosis in preclinical models of liver fibrosis [21,22]. A novel BRD4 inhibitor ZLD-2218 was recently reported to significantly ameliorate kidney fibrosis in a unilateral ureteral obstruction model, by blocking Smad3-mediated transcription of collagen and α-SMA [81]. These examples underscore that targeting scaffolds in the TGF-β axis—whether at the membrane, cytosol, or chromatin level—can yield potent antifibrotic effects.

Inhibiting Inflammatory Scaffolds: Several therapies have aimed at scaffold proteins that mediate inflammation and fibrogenesis. Galectin-3 inhibitors are one example already in clinical trials (for example, belapectin in NASH fibrosis). A novel inhibitor derived from a plant polysaccharide, XHH2 (a rhamnogalacturonan-I oligosaccharide), was shown to bind Galectin-3 and disrupt its ability to scaffold cell surface integrins. In a mouse model of toxic liver fibrosis (CCl4-induced), oral administration of XHH2 disrupted the Gal-3/integrin β1 complex in hepatic stellate cells, thereby suppressing HSC activation and reducing collagen deposition [82]. XHH2-treated mice had significantly lower fibrosis scores without obvious toxicity, and effective doses were as low as 2 mg/kg. This demonstrates a feasible antifibrotic therapy targeting an extracellular scaffold (Gal-3) to prevent it from clustering profibrotic signaling complexes.

Modulating GPCR-Linked Scaffolds: G protein-coupled receptor (GPCR) signaling contributes to fibrosis (e.g., angiotensin II, endothelin, chemokine receptors). β-arrestins, which are scaffolds linking GPCRs to MAPK pathways, have been implicated in cardiac fibrosis and heart failure. A traditional Chinese medicine formulation, Xinshuibao (XSB), was shown to downregulate β-arrestin-1 in a rat model of heart failure, which correlated with reduced fibrosis markers and improved cardiac function [83]. While the exact components of XSB are complex, this result suggests that pharmacological modulation of β-arrestin scaffolds can alter cardiac remodeling. Future drugs might target β-arrestin interactions more specifically (for instance, “biased ligands” at GPCRs that drive signaling away from β-arrestin-mediated pathways). Another GPCR-related target is CXCR4, a chemokine receptor whose signaling fosters fibrosis via recruitment of fibrocytes and activation of AKT/mTOR. Although CXCR4 itself is a receptor, a unique therapeutic called AD-114 was developed as an “i-body” (single-domain antibody) that binds and blocks CXCR4. AD-114 and its optimized version AD-214 effectively reduced kidney fibrosis in multiple models, lowering macrophage infiltration and collagen deposition [84]. By inhibiting CXCR4, these agents indirectly prevent scaffold proteins like β-arrestin and others from being engaged, thereby interrupting downstream profibrotic signaling. AD-214 is notable for being a biologic with extended half-life, showing that advanced protein therapeutics are being pursued in fibrosis beyond traditional small molecules. (CXCR4 targeting is slightly tangential to scaffold proteins, but it showcases how disrupting a receptor can influence scaffold-mediated pathways like AKT/mTOR.)

Targeting Mechanotransduction Complexes: Because mechanotransduction amplifies fibrosis, therapies have aimed at components like TRPV4. A small-molecule TRPV4 inhibitor (GSK2193874) was tested in bleomycin lung fibrosis models and found to reduce lung inflammation and fibrosis by blocking TRPV4–mediated Ca^2+^ influx in fibroblasts, which is necessary for the TRPV4–PI3Kγ scaffold function. Similarly, inhibitors of PI3Kγ (such as AS605240) have attenuated fibrosis in these models, essentially breaking the TRPV4–PI3Kγ signaling axis [27]. FAK inhibitors (e.g., defactinib) target focal adhesion signaling (though FAK is an enzyme, its inhibition disrupts focal adhesion scaffold assembly). These have shown antifibrotic effects in lung and liver models, and some are in early clinical trials for IPF. Another approach is using molecules that soften the ECM or prevent its stiffening (like LOX inhibitors); indirectly, this reduces the engagement of mechanotransduction scaffolds.

Nuclear Scaffold Disruptors and Epigenetic Therapies: We discussed BRD4 inhibitors above. Another nuclear scaffold, PML, could be targeted by drugs like arsenic trioxide (ATO), which induces PML degradation. ATO is already used in leukemia to target PML and could be repurposed for fibrosis if carefully dosed, as it might dismantle PML nuclear bodies that propagate TGF-β signaling. Additionally, HDAC inhibitors and HAT inhibitors (which alter chromatin acetylation) can indirectly affect scaffold recruitment to chromatin (for example, reducing BRD4 binding by lowering histone acetylation). These epigenetic drugs have shown some antifibrotic promise in preclinical studies, although their broad effects pose challenges.

In summary, a wide array of therapeutic modalities are being explored to target scaffold proteins in fibrosis. We have summarized many of these interventions, ranging from small molecules (JQ1 for BRD4, integrin antagonists) and peptides (CSP7 for Cav1, cell-penetrating peptides for MyD88) to biologics (Gal-3 inhibitors, i-body AD-214 for CXCR4). The unifying theme is that disrupting a single scaffold protein can have outsized effects on a network of signals, making it an efficient way to modulate complex pathological processes like fibrosis. However, specificity is key; since scaffold proteins often have roles in normal physiology, therapies must aim to selectively inhibit the pathological interactions while sparing the normal functions. This remains a significant challenge. Nonetheless, the success seen in preclinical models gives hope that one or more of these scaffold-targeted strategies will translate into effective treatments for patients with fibrotic diseases.

## 6. Conclusions

Scaffold proteins have emerged as essential orchestrators of the signaling specificity and crosstalk that drive fibrosis. By serving as platforms for assembling signaling complexes, these proteins ensure that pathways like TGF-β, immune/inflammatory cascades, Wnt, and mechanotransduction are activated in a coordinated manner in time and space. The evidence reviewed here highlights that scaffold proteins are not mere passive organizers, but active regulators that can amplify or dampen signaling outputs. Fibrosis research is increasingly recognizing that understanding these “signal integrators” is crucial for deciphering disease mechanisms. However, many questions remain about the precise mechanisms by which scaffold proteins control signaling dynamics in living tissues. Cutting-edge methods—such as real-time fluorescence resonance energy transfer (FRET) imaging of protein interactions, single-cell RNA/protein sequencing to see scaffold expression in specific cell subpopulations, and computational modeling of signaling networks—will be indispensable for capturing the transient and context-dependent roles of scaffold proteins in fibrosis. Integrating these approaches with traditional molecular biology will help map the complex scaffold interactomes under fibrotic conditions and possibly reveal new scaffold proteins that have so far been overlooked.

From a therapeutic standpoint, scaffold proteins present both an opportunity and a challenge. Their unique position at nodal points of signaling makes them attractive targets to modulate entire pathways. As we have seen, interfering with one scaffold can simultaneously affect multiple downstream fibrogenic signals (for instance, blocking Peli1 can reduce NF-κB, inflammasome, and paracrine signaling all at once). However, because scaffold protein interactions are often transient and multipartner, developing drugs that precisely target these interfaces without off-target effects is difficult. There is also the risk of disrupting normal cellular functions, given that many scaffolds (like integrins or Cav1) play important roles in homeostasis and tissue repair. Therefore, a key goal is to achieve context specificity, to inhibit a scaffold’s role in pathological fibrosis while sparing its role in physiological processes. This might be addressed by targeted delivery systems (such as fibroblast-specific nanoparticles, e.g., the VCAM1-targeted nanocarrier), or by designing allosteric modulators that only exert effects under the altered conditions of diseased cells (e.g., high ROS, stiff matrix). High-throughput screening and structure-guided design of inhibitors for protein–protein interactions are rapidly advancing, offering hope that “undruggable” scaffold proteins can indeed be drugged.

Finally, it is worth noting the tissue-specific aspects of scaffold protein function. As discussed, some scaffolds exhibit different (even opposite) roles in different organs or cell types, like Cav1 being protective in one context and detrimental in another, or Numb promoting Wnt signaling in lung epithelium but aiding autophagy in kidney epithelium. This calls for a nuanced, context-specific approach in any future therapies. Personalized or precision medicine for fibrosis may involve identifying which scaffold-driven pathway is dominant in a given patient’s disease (for example, a patient with an autoimmune hepatitis leading to fibrosis might benefit more from targeting inflammatory scaffolds, whereas another with idiopathic pulmonary fibrosis might need a therapy targeting mechanotransduction scaffolds). Therefore, continued research to delineate scaffold protein networks in specific pathologic contexts (heart vs. lung vs. liver fibrosis, early- vs. late-stage disease, etc.) is essential. Such insights will pave the way for novel therapeutic interventions that are both effective and safe, potentially transforming the management of fibrotic diseases which are currently often progressive and fatal. The convergence of bioengineering, computational network analysis, and pharmacology in the study of scaffold proteins holds promise to finally “untangle” fibrosis’s Gordian knot of signaling and intervene in its course.

## Figures and Tables

**Table 1:** Scaffold proteins in major fibrotic signaling pathways.

Pathway	Scaffold Protein	Function in Fibrosis	Key Mechanism	Therapeutic Potential
TGF-β	Integrins (α6β) [1]	Activates latent TGF-β	ECM mechanosensing	Integrin inhibitors (e.g., STX-100)
	Hic-5 (TGFB1I1) [2,3,4]	Amplifies TGF-β/Smad signaling	Sequesters Smad7, enhances ECM stiffness	ASOs (antisense oligonucleotides) against Hic-5
	Ski [5,6]	Suppresses TGF-β signaling	Smad co-repressor	Overexpression to block fibrosis
Inflammatory	Peli1 [7]	Promotes inflammatory and fibrotic signaling	E3 ligase/adaptor facilitating TAK1/IKK activation (NF-κB/AP-1) and exosome-mediated cell communication	Peli1 inhibitors to block its ubiquitin ligase activity
	MyD88 [8,9]	Links TLR to NF-κB	Myddosome formation	TLR/MyD88 inhibitors
	Bcl10 [10,11]	NF-κB activator	CBM complex scaffold	Bcl10 inhibitors
	Galectin-3 [12]	Enhances inflammasome activation	mitochondrial damage, ASC oligomerization, and NLRP3 inflammasome assembly	Galectin-3 inhibitors (e.g., belapectin)
Wnt/β-Catenin	Axin1 [13,14]	Represses β-catenin	Forms β-catenin destruction complex	Tankyrase inhibitors (Axin stabilizers)
	Dishevelled (Dvl) [13]	Amplifies Wnt signaling	Scaffolds receptor signaling	Dvl polymerization inhibitors
Mechanotransduction	Caveolin-1 (Cav1) [15,16,17]	Organizes focal adhesion signaling	Regulates ECM stiffness, TGF-β activation	Cav1 peptides (CSP7)
	TKS5 [18,19]	Promotes fibroblast invasion	Forms podosomes for ECM degradation	TKS5 inhibitors
Other Pathways	BRD4 [20,21,22]	Sustains fibrotic transcription	Scaffolds super-enhancers	BET inhibitors (e.g., JQ1, ZLD-2218)
	AKAP2 [23,24]	Dual role in cAMP/PKA and actin dynamics	Integrates ERK1/WAVE2 in myofibroblasts	Targeted AKAP2 inhibition in fibroblasts

**Table 2:** Organ-specific scaffold proteins in fibrosis.

Organ	Scaffold Protein	Role in Fibrosis	Mechanism
Lung	IQGAP1 [25,26]	Modulates TGF-β/Wnt signaling	Scaffolds β-catenin and actin regulatory complexes
	TRPV4–PI3Kγ [27,28]	Mediates ECM mechanotransduction	Couples ECM stiffness sensing to AKT activation
Liver	FLNA [29]	Enhances pro-inflammatory and fibrogenic responses	Links TLR4 with STAT3 in macrophages/HSCs, thereby increasing cytokine (e.g., IL-6, TNF-α) and TGF-β production
	AKAP12 [30,31,32]	Suppresses fibrotic progression	Inhibits PI3K/AKT and NF-κB inflammatory pathways
Heart	AKAP2 [24]	Promotes cardiac fibroblast migration and contraction	Functions as a scaffold to assemble the ERK1/WAVE2 complex, regulates cytoskeletal reorganization, and enhances fibroblast invasion and migration
	SHARPIN [33]	Amplifies TGF-β signaling in fibrosis	Stabilizes TGF-β receptor complex assembly
Kidney	PDZK1 [34]	Enhances antioxidant uptake, reduces oxidative stress	Acts as a scaffold regulating OCTN1 transporter to promote dietary antioxidant uptake and mitigate ROS-induced damage
	SPAG9 [35]	Promotes antifibrotic autophagy	Scaffolds Beclin-1 and MAPK signaling modules

**Table 3:** Targeted therapeutic approaches and clinical trial agents in fibrosis.

Scaffold Target	Therapeutic Agent/Approach	Mechanism of Action	Clinical Trial Agents
Integrins (α6β1, αVβ6)	Blocking antibodies	Inhibits mechanical activation of latent TGF-β and downstream signaling	STX-100 (failed in IPF trials)
Caveolin-1 (Cav1)	CSP7 peptide	Disrupts pathological Cav1 interactions; restores alveolar epithelial cell autophagy	LTI-2355 (preclinical); LTI-03 in Phase 1 for IPF
BRD4	INHAL-101; ZLD-2218	Disassembles profibrotic super-enhancers by blocking BRD4-acetylated chromatin binding	ZLD-2218 for kidney fibrosis (preclinical); INHAL-101 for IPF (application)
Galectin-3	Belapectin; GB-1107	Blocks receptor clustering and inflammasome assembly; reduces pro-inflammatory cytokines	Belapectin (Phase 3 for NASH) GB-1107 preclinical for IPF
MyD88	MyD88 inhibitory peptides	Inhibits Myddosome assembly and NF-κB/MAPK signaling	ST-2825 (preclinical for inflammatory diseases but fibrotic diseases)
Bcl10	CBM complex disruptors	Attenuates NF-κB activation by blocking CARMA–Bcl10–MALT1 assembly	Phase 1 but in lymphoma
Peli1	Peli1 inhibitors	Inhibits TAK1/IKK-NF-κB/AP-1 signaling and exosome-mediated communication	BBT-401 Phase 1 but in ulcerative colitis
CXCR4	AD-114/AD-214 (i-body therapy)	Blocks fibrocyte recruitment and macrophage infiltration; inhibits AKT/mTOR signaling	None reported in fibrosis
Focal Adhesion Kinase (FAK)	Defactinib	Disrupts focal adhesion complexes to block mechanosensitive fibrotic activation	Reported in cancer but not in fibrosis
PML	Arsenic trioxide (ATO)	Degrades PML nuclear bodies to suppress TGF-β/p53-driven fibrosis genes	ATO (approved for leukemia)

## Data Availability

Not applicable.
